# Controlling Nutritional Status Score Before Receiving Treatment as a Prognostic Indicator for Patients With Urothelial Cancer: An Exploration Evaluation Methods

**DOI:** 10.3389/fonc.2021.702908

**Published:** 2021-10-13

**Authors:** Lei Peng, Chunxiao Du, Chunyang Meng, Jinze Li, Chengyu You, Xianhui Li, Pan Zhao, Dehong Cao, Yunxiang Li

**Affiliations:** ^1^ Department of Urology, Nanchong Central Hospital, The Second Clinical College, North Sichuan Medical College (University), Nanchong, China; ^2^ Department of Clinical Pharmacy, Sichuan Cancer Hospital & Institute, Sichuan Cancer Center, School of Medicine, University of Electronic Science and Technology of China, Chengdu, China; ^3^ Department of Urology, Institute of Urology, West China Hospital, Sichuan University, Chengdu, China; ^4^ State Key Laboratory of Biotherapy and Cancer Center, West China Hospital, Sichuan University, Collaborative Innovation Center for Biotherapy, Chengdu, China

**Keywords:** controlling nutritional status, urothelial cancer, bladder cancer, upper tract urothelial carcinoma, meta-analysis, prognostic factors

## Abstract

**Introduction:**

This meta-analysis aims to assess whether the Controlling nutritional status (CONUT) score before treatment can be an independent predictor of the prognosis of patients with urothelial cancer (UC).

**Methods:**

The system searches Web of Science, PubMed, MEDLINE, China National Knowledge Infrastructure (CNKI), and Cochrane Library, and the search time is up to April 2021. Use STATA 16.0 and Engauge Digitizer 4.1 software for data processing and statistical analysis.

**Results:**

A total of 8 studies were included in this meta-analysis. The meta-analysis results show that compared with the low CONUT group, the high CONUT group has worse over survival (OS) [HR=1.58, 95%CI (1.34, 1.86), P=0.001], cancer-specific survival (CSS) [HR=2.03, 95%CI (1.25-3.29), P=0.04] and recurrence-free survival (RFS) [HR=1.97, 95%CI (1.15, 3.40), P=0.014]; for progression-free survival (PFS), or disease-free survival (DFS), the difference between the two groups was not statistically significant [HR=2.30, 95%CI (0.72, 7.32), P=0.158]. According to different carcinoma types, cut-off value, and region, subgroup analysis of OS was performed, and similar results were obtained.

**Conclusions:**

Based on current evidence, this meta-analysis proves that the CONUT score of UC patients before treatment is an independent prognostic predictor. It performs well on OS, CSS, and RFS, but the conclusions on DFS/PFS need to be treated with caution.

**Systematic Review Registration:**

https://www.crd.york.ac.uk/prospero/display_record.php?ID=CRD42021251890, identifier CRD42021251890.

## Introduction

Urothelial cancer (UC) mainly refers to cancer of bladder cancer (BC), upper tract urothelial cancer (UTUC), and other organs covering the transitional epithelium of the urinary tract (1). BC is the tenth most common cancer in the world. In 2020, there will be about 573,000 new BC cases worldwide, and about 213,000 people will die from it. From an epidemiological perspective, men are more likely to suffer from BC than women. It is the sixth most common malignant tumor in men and the ninth leading cause of death from carcinoma (2). The standard treatment for non-muscular invasive bladder cancer is periodic adjuvant chemotherapy after transurethral resection of bladder tumor (3). In patients with muscle-invasive bladder cancer (MIBC), only chemotherapy is less effective, and radical cystectomy is the accepted treatment for MIBC (4). Any pathological type of bladder cancer has a recurrence, fatal complications, and poor prognosis after treatment. At present, a lot of studies have proved that some genes, the ratio of fibrinogen to serum albumin, the percentage of neutrophils to lymphocytes, and the modified Glasgow Prognostic Score (GPS) can be used to assess the prognosis of BC (5–7). UTUC includes ureteral cancer and renal pelvic cancer, with a low incidence, accounting for less than 10% of urothelial cancers. However, compared with other genitourinary system cancers, it has a higher degree of malignancy and a worse prognosis (8). The five-year survival rate of patients with myometrial infiltrating UTUC after surgery is low, and the abysmal prognosis and recurrence bring physical and psychological damage to the patients (9, 10). Tumor pathology data obtained from surgical specimens or biopsy is an indicator for predicting the prognosis of patients, but not every UC patient can receive surgical treatment or undergo radical surgery (11). Practical evaluation and prediction of the patient’s prognosis based on the laboratory examination data before surgical treatment are particularly important for disease management, prolonging the patient’s life, and improving the patient’s quality of life.

The proliferation of malignant tumors determines that the damage to human nutrition is severe (12).

In this context, some nutrition-related indicators such as the prognostic nutritional index (PNI) are used to predict the surgical tolerance and prognosis of patients with different types of malignant tumors (13, 14). Controlling nutritional status (CONUT) score is a composite index that combines the subjects’ serum albumin level, total lymphocyte count, and cholesterol level to quantify (15). In recent years, CONUT has been proven to be an independent prognostic factor of cancer and has played a role in treating various cancers such as gastrointestinal cancer, liver cancer, and breast cancer (16–18). The role of CONUT as a predictor of prognosis in genitourinary system tumors, especially in UC, although some studies have been published, there is no unified conclusion (19). The purpose of this study is to evaluate whether the CONUT of patients before treatment is capable of being an independent prognostic factor by including related published studies for systematic reviews and meta-analysis, to assist clinicians in the routine evaluation of patients, and improve the prognosis of UC patients.

## Methods

### Literature Search and Eligibility Criteria

System search is used to find research published in Web of Science, PubMed, MEDLINE, China National Knowledge Infrastructure (CNKI), and Cochrane Library. The time range of the literature search is from the establishment of the database to April 2021. Use CONUT, urothelial carcinoma, upper tract urothelial carcinoma, bladder cancer, and other terms to search for all studies where the above fields appear in the title, abstract, and anywhere else. Besides, some research references were searched manually.

Use the following search fields: “urothelial cancer”, “upper tract urothelial cancer”, “ureter cancer”, “bladder cancer”, “radical cystectomy”, “radical nephroureterectomy”, “treatment”, “malignant tumor”, “Control Nutritional Status”, “CONUT”, “Predictive Factors”, “prognostic indicators”. Randomly assemble the above fields, and replace proper nouns with upper and lower meaning words.

The inclusion and exclusion of the study were as follows: (1) urothelial cancer (including upper urothelial cancer and bladder cancer) was pathologically diagnosed, and there were no other types of malignant or metastatic cancer; (2) before treatment, the patients were graded according to the standard CONUT rating scale (**Table 1**). (3) all patients received systematic treatment for UC (surgery, radiotherapy, chemotherapy, or supportive treatment). (4) the researchers followed up the patients for a certain period and calculated at least one of the over survival (OS), cancer-specific survival (CSS), recurrence-free survival (RFS), progression-free survival (PFS), or disease-free survival (DFS). (5) the effects of the low CONUT group and high CONUT group on the prognosis of surgical patients were discussed, and the hazard ratio (HR) was provided. (6) the design type of the study is a retrospective study and prospective study. (7) the research, which is rated as high quality by the study quality evaluation system. Also, repetitive studies, letters, case reports, reviews, studies unrelated to the subject matter, experimental results from computer models, animal experiments, and theoretical experiments, and studies that could not extract available data were excluded.

**Table 1:** Definition of CONUT score.

Parameters	Normal	Light	Moderate	Severe
Serum albumin (g/dL)	3.5–4.5	3.0-3.49	2.5–2.99	<2.5
Sore	1	2	4	6
Total lymphocyte (count/mm3)	≥1600	1200–1599	800–1199	<800
Sore	0	1	2	3
Total cholesterol (mg/dL)	>180	140–180	100–139	<100
Sore	0	1	2	3
CONUT score (total)	0–1	2–4	5–8	9–12

According to the above inclusion and exclusion criteria, this procedure was independently completed by two researchers (JZ. L and CY. M), and the literature quality was evaluated, and the required data were extracted. The differences that occurred in the process were resolved through negotiation.

### Quality Evaluation

Based on the preliminary search results, the Newcastle-Ottawa Scale (NOS) was used to evaluate the quality of the included studies (20). According to the evaluation of the three question areas of selection, comparability, and exposure in the scale, a score of more than six stars can be considered as high-quality research.

### Data Extraction

The researchers used standard tables to extract the following data for each included study: first author’s name, publication year, study design, sample size, treatment intervention, age, cancer type, cut-off, follow-up time, survival statistics (OS, CSS, RFS, DFS, PFS), HR and 95% confidence intervals (95%CI) about the univariate/multivariate analysis of the low CONUT group ref. High CONUT group.

### Statistical Analysis

Stata 16 (StataCorp LP, University City, Texas, USA) was used for statistical analysis. The HR and its 95% CI of the univariate/multivariate analysis in each study were extracted and statistically combined to assess the importance of the CONUT score for the prognosis of UC patients. Q test and chi-square tests were used to verify the heterogeneity between the included studies. If I^2^ > 50%, the differences between the literature were considered significant (21). According to the test results of heterogeneity, I^2^ ≥ 50% or P < 0.1, and the random-effect model was used to pool estimates. The fixed-effect model is utilized in the opposite situation. According to the region, cut-off value, and carcinoma type for subgroup analysis. In addition, some of the original studies only provided survival curves, so survival data could not be directly obtained. Engauge Digitizer 4.1 software was used to process the survival curve and estimate the survival rate.

## Results

### Description of Studies

**Figure 1:**
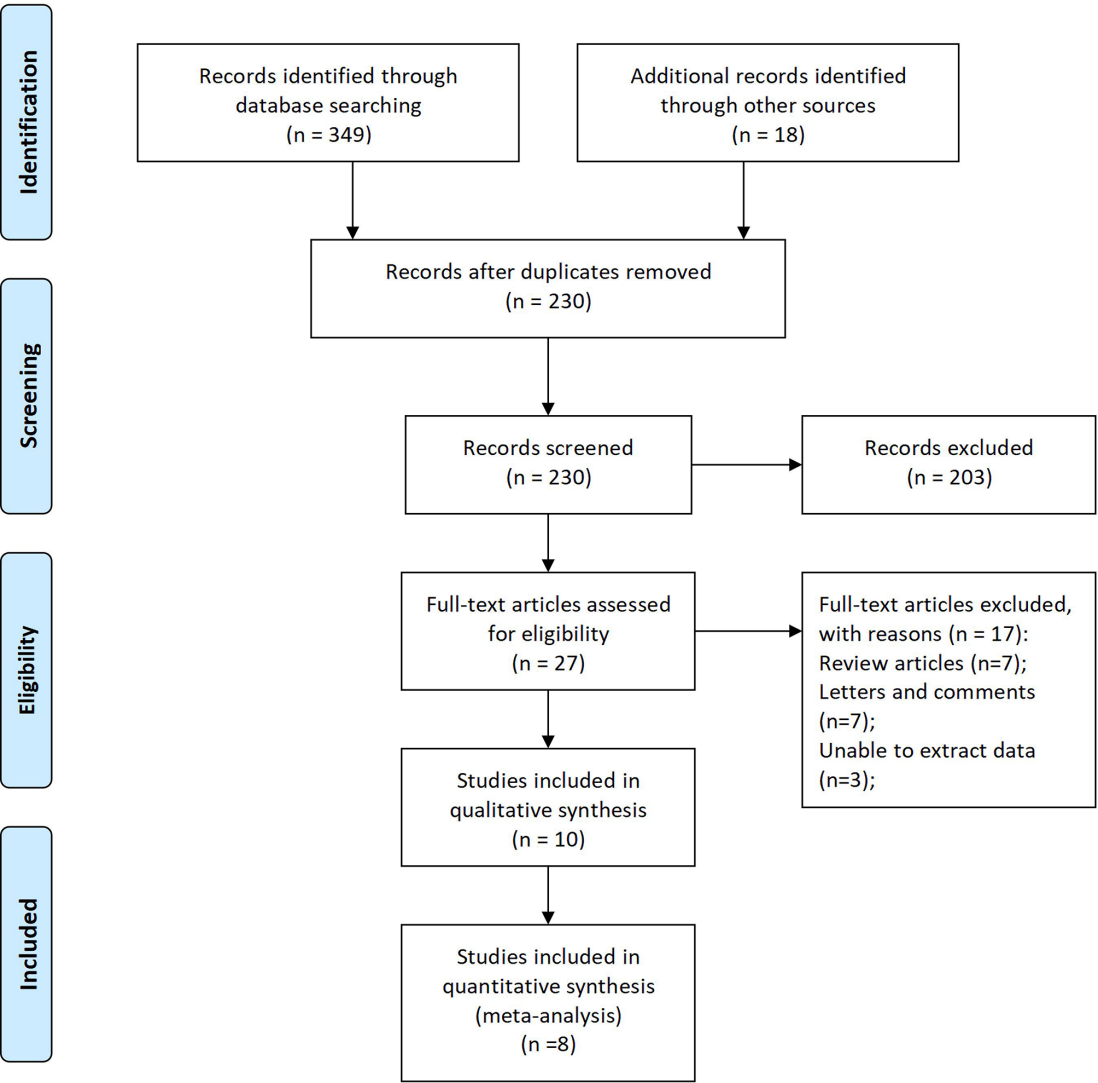
Flow diagram of studies selection process.

349 records were retrieved from 6 databases, and 18 records were manually retrieved from reference citations of related studies. After reading the title and the name of the first author, 137 duplicate studies were excluded. Further analysis of the research topics, abstract, and keywords, 203 records unrelated to the research topics were removed. Full-text analysis of the remaining 27 studies. Excluded from 7 reviews, 7 letters, and comments, 3 studies were unable to extract data entirely, and 2 studies with low literature quality scores. 7 retrospective studies and 1 propensity matching scoring study with 2232 patients were included in this meta-analysis at last (**Figure 1**) (22–29).

The baseline data, including the first author’s name, year of publication, sample size, intervention model, tumor type, follow-up time, and survival index of each study were recorded in **Table 2**. Grouping according to CONUT score, OS, CSS, RFS, DFS, PFS, HR are recorded in **Table 3**.

**Table 2:** Baseline data for studies included in the meta-analysis.

Author, year	Region	Study design	Sample Size	Intervention	Age^a^	Cancer Type	Cut-Off	Follow-Up Time^h^	Outcome Indicators	Quality Score^n^
29	China	R^o^	96	TURBT^b^	60.37 ± 12.49	BC^f^	2	(24-60) month	OS^i^, PFS^j^	6
26	Japan	R	115	RC^c^	69.4 ± 9.4	BC	3	21month (4-61)	OS, CSS^k^, RFS^l^	7
22	China	PSM^p^	754	RC	69 (61-74)	UTUC^g^	3	61month (45-105)	OS, CSS, DFS^m^	8
18	Japan	R	185	Mixed^d^	71 (63-80)	BC, UTUC	2	12.3 month	OS	6
28	China	R	662	RNU^e^	67 (59-74)	UTUC	2	41month	OS, CSS, RFS	8
24	Japan	R	107	RNU	74 (63-85)	UTUC	3	43month (7-79)	OS, CSS, RFS	7
23	China	R	189	RC	68.13 ± 10.61/62.98 s ± 10.84	BC	3	45month(57-81)	OS	6
25	Japan	R	124	RC	72 (61-77)	BC	1	22month (10-64)	OS, CSS	6

a Age, Mean ± SD/Mean (Range).

b TURBT, Transurethral resection of bladder tumor.

c RC, Radical cystectomy.

d Mixed, Radical cystectomy, Radical nephroureterectomy, Chemotherapy, Radiotherapy.

e RNU, Radical nephroureterectomy.

f BC, Bladder Cancer.

g UTUC, Upper tract urothelial carcinoma.

h Follow-up Time, Median (Range)/Median.

i OS, Over Survival.

j PFS, progression- free survival.

k CSS, Cancer-specific survival.

l RFS, Relapse-free survival.

m DFS, Disease-free survival.

n Quality Score, Score based on NOS scale.

o R, Retrospective.

p PSM, Propensity score match.

**Table 3:** Survival statistics for studies included in the meta-analysis.

Author, year	Cohort	Results of Low CONUT/High CONUT				Low CONUT Ref. High CONUT (HR 95%CI)			
		OS	CSS	RFS	DFS/PFS	OS	CSS	RFS	DFS/PFS
(29)	CONUT<2(n=53) CONUT ≥2(n=43)	89.29%*vs.*37.50%	NA^a^		80.64% *vs.* 48.39%	4.503 (1.264-16.037)	NA	4.728 (1.512-12.783)	NA
(26)	CONUT<3(n=22) CONUT ≥3(n=93)	74.6% *vs.* 55.0%	78.7% *vs.* 48.1%	61.1% *vs.* 57.4%	NA	3.83 (1.44-9.56)	6.01 (2.23-16.0)	3.55 (1.41-8.50)	NA
(22)	CONUT<3 (n=550) CONUT≥3 (n=204)	78.0% *vs.*65.0%	82.0% *vs.*70.0%	NA	76.0% *vs.* 60.0%	1.273 (0.960-1.686)	1.328 (0.954-1.847)	NA	1.418 (1.132-1.776)
(18)	CONUT<2(n=91) CONUT ≥2(n=94)	12.08% *vs.* 4.25%		NA		1.57 (1.06-2.31)		NA	
(28)	CONUT<2 (n=270) CONUT ≥2 (n=392)	66.5% *vs.* 46.9%	72.6% *vs.* 53.3%	58.5% *vs.* 42.9%	NA	1.58 (1.18-2.11)	1.69 (1.21-2.34)	1.43 (1.10-1.86)	NA
(24)	CONUT<3 (n=83) CONUT ≥3 (n=24)	66.8% *vs.* 26.4%	71.7% *vs.* 28.1%	66.0% *vs.* 50.1%	NA	2.90 (1.18-6.75)	5.44 (1.95-14.8)	2.26 (0.97-4.94)	NA
(23)	CONUT<3(n=99) CONUT ≥3(n=90)	86.78% *vs.* 58.53%		NA		2.791 (1.258-6.190)		NA	
(25)	CONUT<1(n=53) CONUT ≥1(n=64)	65.8%^b^	71.69%^b^	NA		1.1 (0.5-2.1)	1.1 (0.6-2.9)	NA	

a NA, Not available.

b survival rate of the overall sample is recorded only.

### Quality Assessment

Based on the NOS scoring rules, we have listed the final study quality scores in **Table 2**.

### Survival Outcomes

**Figure 2:**
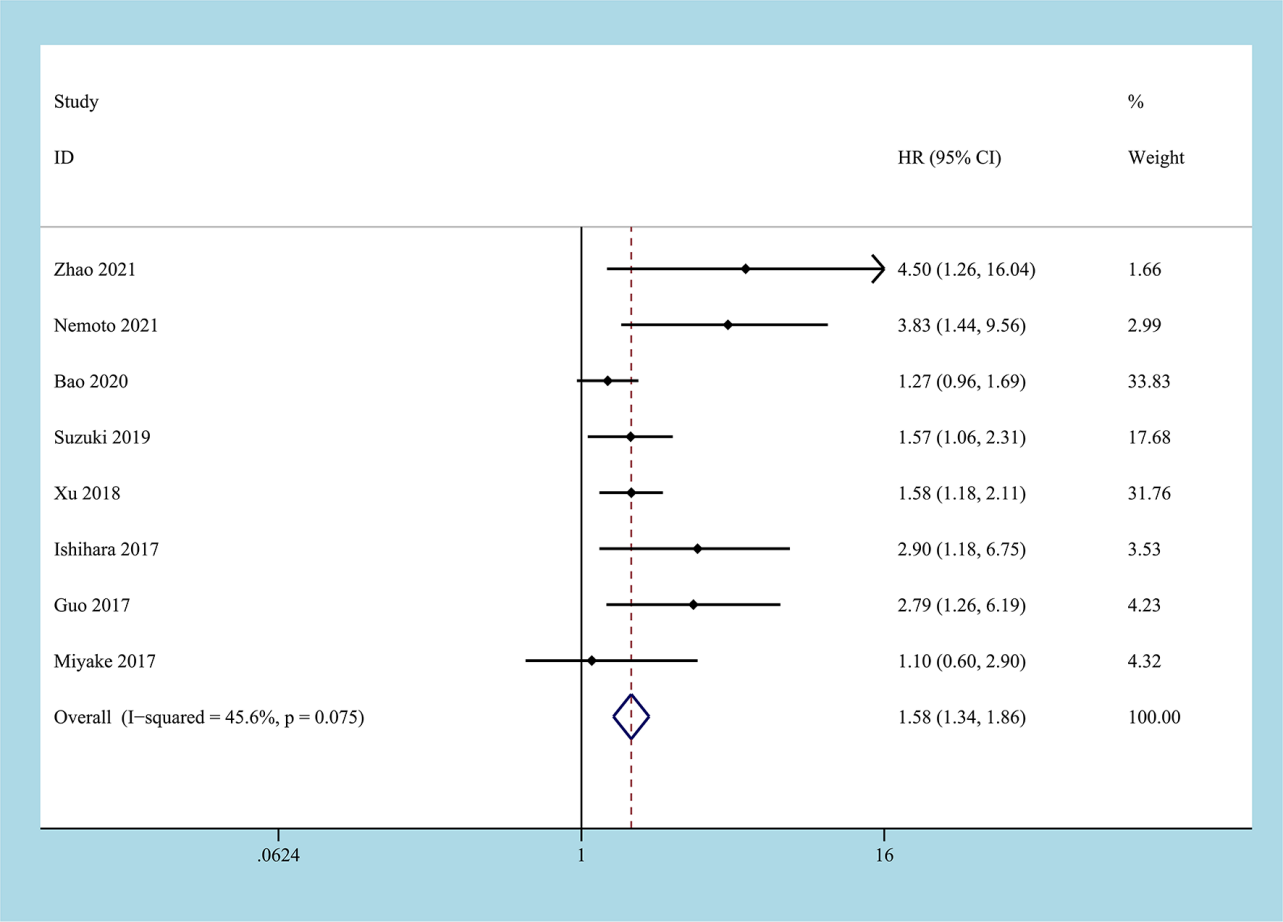
Forest plot and meta-analysis of the relationship between OS and CONUT score.

8 studies reported the HR of the low CONUT group and the high CONUT group on OS, and a total of 2232 patients were enrolled (22–29). After the heterogeneity test, the heterogeneity among the studies was within the acceptable range (I^2^ = 45.6%, P=0.075), and the fixed-effects model was used to pool HR. The results of the meta-analysis indicated that compared with the low CONUT group, the high CONUT group had worse OS, the CONUT score was positively correlated with the worse OS, and the difference between the two groups was statistically significant [HR=1.58, 95%CI (1.34, 1.86), P=0.001] (**Figure 2**).

**Figure 3:**
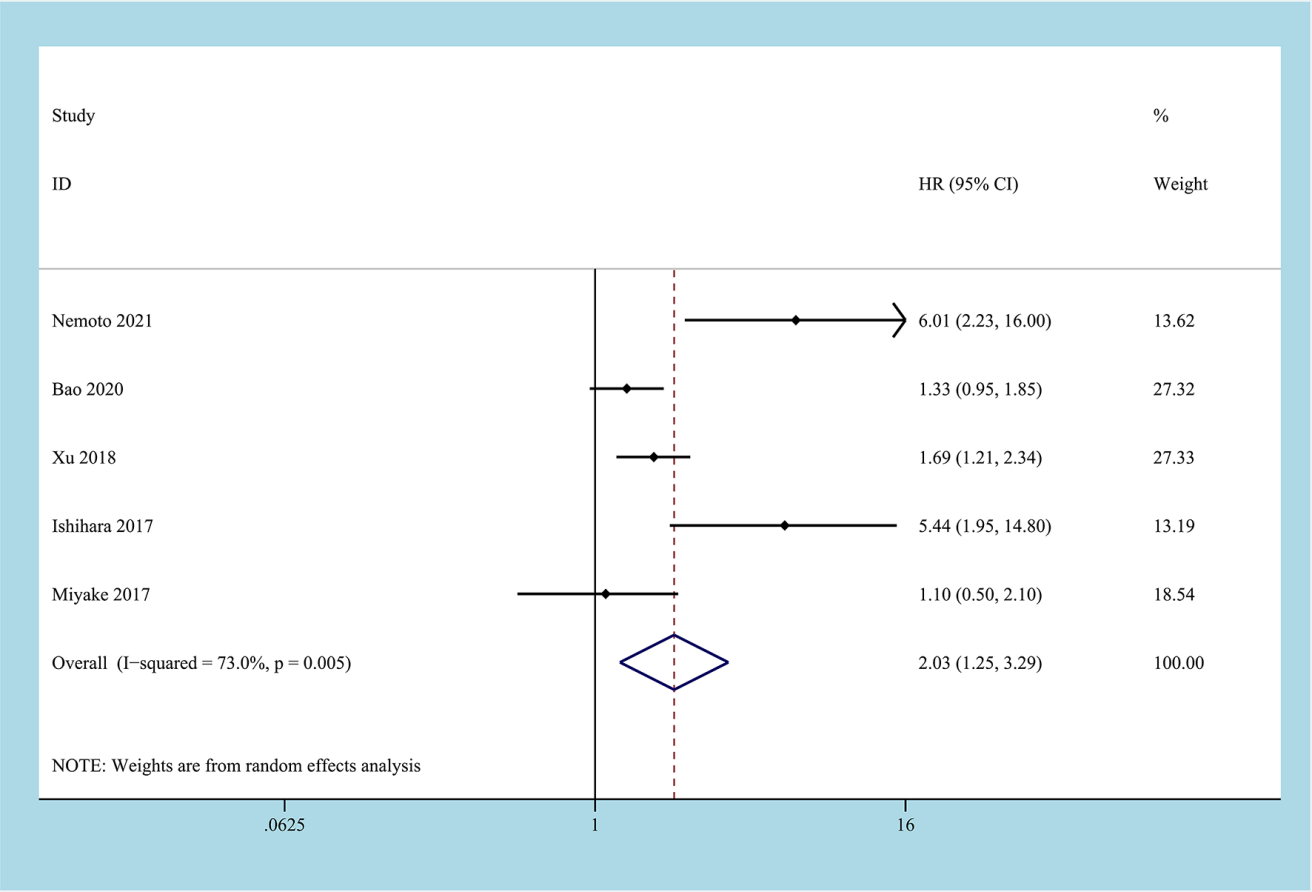
Forest plot and meta-analysis of the relationship between CSS and CONUT score.

There are 5 studies reported on CSS, involving 1762 patient samples (22, 24–26, 28). The heterogeneity test results suggest that the heterogeneity among the studies is relatively high (I2 = 73.0%, P=0.005), and the random-effects model is applicable. The results of the meta-analysis showed that high CONUT was positively correlated with lower CSS. Compared with the low CONUT group, higher CONUT scores before treatment easily led to worse CSS, and the difference between the two groups was statistically significant [HR=2.03, 95%CI (1.25-3.29), P=0.04] (**Figure 3**).

**Figure 4:**
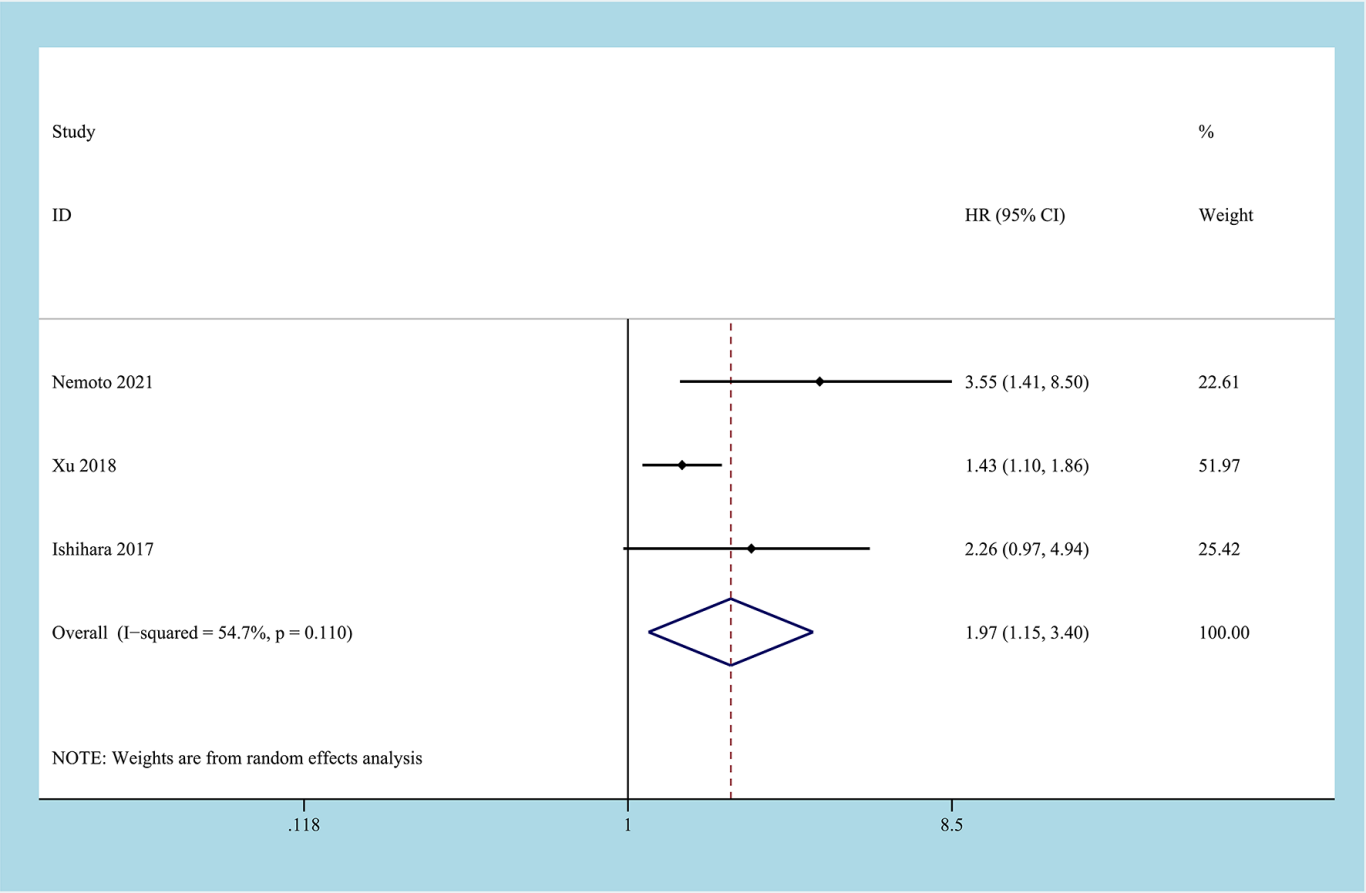
Forest plot and meta-analysis of the relationship between RFS and CONUT score.

3 studies reported the impact of CONUT score on RFS, and a total of 884 patients enrolled (24, 26, 28). Due to the inevitable heterogeneity between the studies (I^2^ = 54.7%, P=0.11), the random-effects model was used to combine the results. The results of the meta-analysis suggested that high CONUT before treatment predicted a worse RFS outcome, and there was a statistical difference between the two groups [HR=1.97, 95%CI (1.15, 3.40), P=0.014] (**Figure 4**).

**Figure 5:**
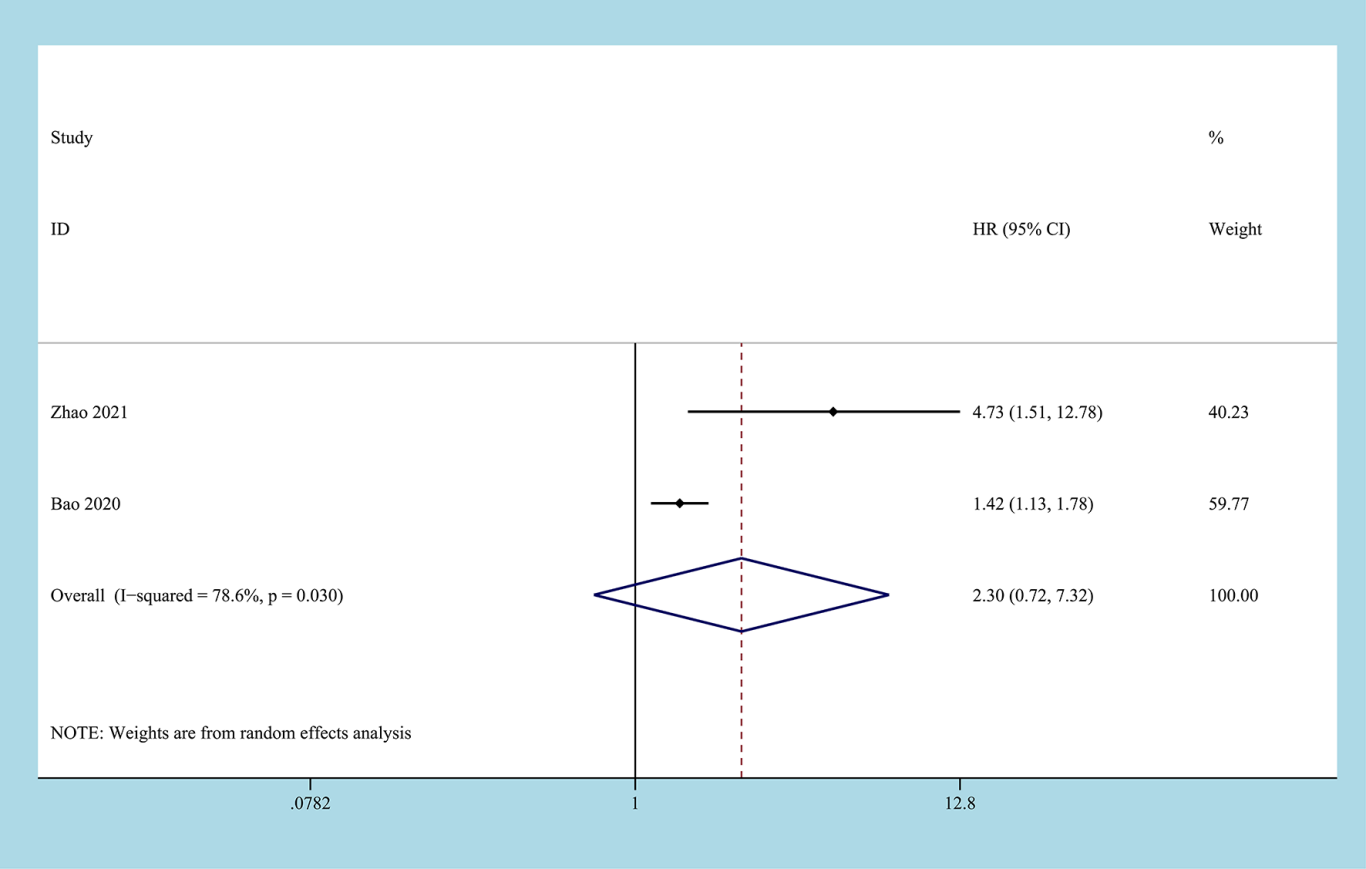
Forest plot and meta-analysis of the relationship between DFS/PFS and CONUT score.

2 studies reported on DFS/PFS, involving 850 patient samples (22, 29). Due to the significant heterogeneity between the studies (I2 = 78.6%, P=0.03), the random-effects model was used to combine the results. According to the results of the meta-analysis, there was no correlation between the CONUT score before treatment and DFS/PFS, and the difference between the two groups was not statistically significant [HR=2.30, 95%CI (0.72, 7.32), P=0.158] (**Figure 5**).

### Subgroup Analysis

According to the carcinoma type, cut-off value, and region, the studies are divided into different subgroups. The effect of CONUT score on OS in UC patients is further explored. Stratified according to the carcinoma type, the BC group [HR=2.35, 95%CI (1.50, 3.69), P=0.005] (23, 25, 26, 29), the UTUC group [HR=1.47, 95%CI (1.20, 1.79), P=0.005] (22, 24, 28) and the Mixed group [HR=1.57, 95%CI (1.06, 2.32), P=0.023] (27) all indicated that a high CONUT score was positively correlated with worse OS (**Table 4**). Stratified according to the cut-off value, the results of the meta-analysis suggested that in the cut-off value ≤2 [HR=1.59, 95%CI (1.23, 2.04), P=0.001] (25, 27–29) and >2 groups [HR=2.27, 95%CI (1.24, 4.16), P=0.008] (22–24, 26), a high CONUT score predicted a lower OS. The difference between the two groups was statistically significant (**Table 4**). Stratified by region, the results of the meta-analysis suggest that in China [HR=1.69, 95%CI (1.19, 2.41), P=0.004] (22, 23, 28, 29) and Japan [HR=1.19, 95%CI (1.18, 3.10), P=0.008] (24–27), lower OS is more likely to be related to higher CONUT. Compared with the low CONUT group, the high CONUT group has a worse OS outcome. There are statistical differences between the groups (**Table 4**).

**Table 4:** Subgroup analysis of OS based on different influencing factors.

Subgroups	Cohort	Include study	Effect model	HR (95%CI)	P	Heterogeneity	
						I^2^ (%)	P
**Carcinoma type**	BC^a^	4 [23,25,26,29]	Fixed	2.35 (1.50, 3.69)	0.005	48.1	0.123
	UTUC^b^	3 [22,24,28]	Fixed	1.47 (1.20, 1.79)	0.005	44.0	0.168
	Mixed^c^	1 [27]	Fixed	1.57 (1.06, 2.32)	0.023	NA^d^	NA
**Cut-off value**	≤2	4 [25,27–29]	Random	1.59 (1.23, 2.04)	0.001	12.4	0.331
	<2	4 [22-24.24]	Random	2.27 (1.24, 2.28)	0.008	68.2	0.024
**Region**	China	4 [22,23,28,29]	Random	1.69 (1.19, 2.41)	0.004	54.8	0.085
	Japan	4 [24–27]	Random	1.91 (1.18, 3.10)	0.008	45.9	0.136

a BC, Bladder Cancer.

b UTUC, Upper tract urothelial carcinoma.

c Mixed, Bladder cancer and Upper tract urothelial carcinoma.

d Only one study was included in the subgroup, and the heterogeneity test could not be performed.

## Discussion

CONUT is a composite quantitative index based on serum albumin, total cholesterol, and peripheral blood lymphocyte technology. Exploring the prognostic value of the CONUT score in cancer patients is nothing new. Initially, CONUT was used to evaluate the prognosis of nutritional status before surgery and postoperative complications. Still, in recent years, ongoing studies have shown that CONUT can be used as an independent predictor of the prognosis of a variety of malignant tumors (30, 31). In malignant tumors of the genitourinary system, many researchers have explored the ability of CONUT as an independent predictor, but the opinions are different, and there is no conclusion. This article aims to evaluate the potential of CONUT as an independent predictor of the prognosis of UC patients based on current real-world evidence.

This meta-analysis performed evidence-based medicine analysis on 8 published studies that explored the prognostic and survival indicators of CONUT in UC patients. Our results were in line with these studies, which support the CONUT score as an independent predictor for survival outcomes. The results of this meta-analysis are consistent with the conclusions of most of the included studies and support CONUT as an independent predictor of survival outcomes (24, 26, 28). In particular, compatible with all research conclusions, we have all proved that the CONUT score is an independent factor for OS, CSS, and RFS. However, due to the limitation of the number of studies and the number of cases, the results of the meta-analysis failed to reveal the independent predictive value of CONUT in DFS/PFS.

A subgroup analysis of OS based on different influencing factors yielded similar results. This not only supports the conclusions of this meta-analysis but also provides some enlightenment from it. Carcinoma type is not a factor that limits the realization of CONUT scores. Carcinoma type is not a factor that limits the realization of CONUT scores. From the results of subgroup analysis, in BC and UTUC, the high CONUT group led to poor OS outcomes. This conclusion supplements the gap in the role of CONUT score in genitourinary system tumors. The standard CONUT score sheet will judge a score> 1 as a mild abnormality. A stratified subgroup analysis based on the cut-off value showed that when the cut-off value is greater than 2, the combined HR of the high CONUT group is higher than the cut-off value ≤ 2. However, the subgroup analysis based on the regional stratification can only prove the prognostic, predictive effect of the CONUT score in the two regions, which suggests further research directions.

An ideal cancer predictor should include several elements that are objective, inexpensive, easy to operate, and applicable before surgery. Similar to objective data assessment (ODA) and subjective global assessment (SGA), the CONUT score has been reported to be a cost-effective and straightforward method of comprehensively and objectively detecting and controlling hospital malnutrition (22). Due to the particularity of the CONUT score composition, it reflects the body’s protein metabolism, immune function, and lipid metabolism, and to a certain extent, represents the body’s nutritional status and systemic immune-inflammatory response (32).

Serum albumin represents the nutritional status of the body, and studies have shown that hypoalbuminemia is related to the poor prognosis of cancer; Studies have shown that shorter OS was observed in patients with hypoalbuminemia (5-year OS 17.1%) when compared to patients with normal serum albumin levels (5-year OS 58.6%, p = 0.004) in vulvar cancer (33). Albumin also reflects the inflammation caused by malignant tumors to a certain extent (34). Previous studies have proved that albumin to globulin ratio (AGR), a predictor related to albumin, is an independent predictor of the prognosis of testicular cancer (35). Serum albumin levels are regulated by many factors, including cytokines such as interleukin-6 and tumor necrosis factor-α. In addition, ascites and liver cell damage can also lead to hypoproteinemia (36, 37). These studies proved the role of serum albumin as a nutrient in inflammation and cancer and supported the conclusions of this meta-analysis. Lymphocytes play an anti-tumor effect in the immune system by influencing tumor growth, metastasis, apoptosis, and inducing cytotoxicity in the body. In the case of advanced cancer patients, cancer cells can destroy lymphocytes by editing pro-apoptotic ligands, thereby promoting the immune escape of tumor cells. The anti-tumor immune response mediated by CD8^+^ T lymphocytes plays an essential role in the progression and development of anti-tumor. However, during the development of cancer, it usually suffers from dysfunction due to immune-related tolerance and immunosuppressive effects of the tumor microenvironment. Cancer-related fibroblasts, macrophages, and regulatory T cells may create an immune barrier to the immune function of T cells, resulting in a decrease in the number of T lymphocytes and protecting tumor cells from the damage of the immune response (38). Researcher Templeton and his colleagues demonstrated in their meta-analysis that the ratio of neutrophils to lymphocytes could be used as an independent prognostic predictor of solid tumors (39). Cholesterol is an integral part of the cell membrane, and it is potentially related to tumor cell proliferation, metastasis, and immune response. Cholesterol is an essential part of the cell membrane, and it is potentially related to tumor cell proliferation, metastasis, and immune response (40). At the same time, studies have reported that cholesterol can increase the antigen presentation function of monocytes and accelerate the process of immune cells recognizing tumor cells. This mechanism indirectly affects the immune response of the tumor microenvironment (23). These research conclusions and mechanisms support the excellent performance of the CONUT score as an independent prognostic evaluation factor for UC patients.

The Eastern Cooperative Oncology Group performance score (ECOG-PS) is used to assess a patient’s physical status so that clinicians can understand the patient’s level of current physical ability and activity (41).There were 5 options, which ranged from “I am fully active and able to carry out activities as I did before my cancer diagnosis, without any restriction” to “I am completely disabled, cannot carry on my self-care, and I am confined to a bed or chair (41)”. Research on ECOG-PS as an independent prognostic factor of malignant tumors has been published. Lisa and colleagues evaluated whether ECOG-PS can be used as a predictor of patient prognosis in bladder cancer patients who received the “Organ-preserving Treatment”. The research results showed that patients with ECOG-PS of 0-1 had a significantly better 5-year survival than patients with ECOG-PS of 2-3 (64% *vs.* 0%, p<0.001) (42). In addition, the results of some scholars also showed that in patients with UTUC who underwent radical nephroureterectomy, preoperative ECOG-PS was an independent predictor of CSS (HR=1.89, P=0.019) (43). It should be noted that the evaluation of ECOG-PS is more subjective than the CONUT score objectively derived from the blood test results. For the same subject, the ECOG-PS obtained from the three perspectives of patients, clinicians, and nurses usually has differences in authenticity and accuracy (44, 45). Accurately assess the ECOG-PS of subjects, and its standards need to be further regulated. Studies have shown that clinicians overestimate ECOG-PS compared with patients themselves. The reason is that in addition to physical health, patients also include their social and emotional health into the score. Most clinicians cannot obtain this information immediately from standard patient interviews alone, which may be the reason why clinicians overestimate patients’ ECOG-PS (44). NEEMAN compared the ECOG-PS evaluated by clinicians and nurses on patients, and found that the ECOG-PS score of nurses seems to be more predictive of important results, and the inconsistency of clinicians in ECOG-PS score indicates worse results. The nurse’s score may bring additional clinical benefits (45). Studies have shown that preoperative hypoproteinemia is associated with poor postoperative prognosis of urological malignancies (46). The nutritional status of patients before surgery is closely related to the recovery of patients after surgery. Therefore, the hypoalbuminemia of patients with urothelial carcinoma should be corrected as much as possible before surgery. Multiple single-center studies have shown that preoperative serum albumin level is a predictor of survival after radical cystectomy (47). Compared with patients with normal albumin levels, patients with preoperative albumin levels have significantly lower OS and CSS, and are more likely to have fatal complications (48, 49). However, multi-center studies have proved that when albumin is higher than 4mg/dl, the benefit will be reduced (50). Combining ECOG-PS with preoperative albumin levels to jointly predict the prognosis of urothelial carcinoma is worthy of further study. This combined item takes both subjective and objective factors into consideration, and has considerable potential advantages. Compared with using the CONUT score alone, it has certain advantages, but it can also incorporate more objective indicators for joint evaluation. The more accurately the patient’s basic condition before receiving treatment can be grasped, the more the patient can achieve the maximum benefit after treatment. Based on the current evidence and depth of research, these speculations need further research to prove.

We followed PRISM guidelines strictly to perform this meta-analysis (51). However, some limitations cannot be avoided. At first, the included studies are primarily retrospective, and the level of evidence in evidence-based medicine is not high enough so that the conclusions of this meta-analysis need to be treated with caution. Second, the number of included studies is relatively small. Furthermore, it is observed that the source area of the literature contains only two countries, and the effect of the CONUT score in the population is biased because the research on the relationship between CONUT and UC in European and American countries is not included.

## Conclusion

Based on current evidence, this meta-analysis proves that the CONUT score of UC patients before treatment is an independent prognostic predictor. It performs well on OS, CSS, and RFS, but the conclusions on DFS/PFS need to be treated with caution. This conclusion needs to be verified by a prospective cohort study with larger sample size and a more rigorous design.

## Data Availability Statement

The original contributions presented in the study are included in the article/supplementary material. Further inquiries can be directed to the corresponding authors.

## Author Contributions

They conceived and designed the experiments: YL and DC. Analysed the data: LP, JL, CM. Contributed reagents/materials/analysis: CD, CY, PZ, and XL. Wrote the manuscript: LP and JL. All authors contributed to the article and approved the submitted version.

## Funding

This work was supported by the Sichuan Province Science and Technology Planning Project under Grant number 2020YFS0320; Sichuan Provincial Health Committee Research Project under Grant number 20PJ305.

## Conflict of Interest

The authors declare that the research was conducted in the absence of any commercial or financial relationships that could be construed as a potential conflict of interest.

## Publisher’s Note

All claims expressed in this article are solely those of the authors and do not necessarily represent those of their affiliated organizations, or those of the publisher, the editors and the reviewers. Any product that may be evaluated in this article, or claim that may be made by its manufacturer, is not guaranteed or endorsed by the publisher.

## Abbreviations

CONUT, controlling nutritional status; UC, urothelial cancer; BC, bladder cancer; UTUC, upper tract urothelial cancer; MIBC, muscle-invasive bladder cancer; GPS, Glasgow Prognostic Score; PNI, prognostic nutritional index; CNKI, China National Knowledge Infrastructure; NOS, Newcastle-Ottawa Scale; OS, Over Survival; CSS, Cancer-Specific Survival; DFS, Disease-Free Survival; RFS, Recurrence‐free survival; PFS, progression-free survival; HR, hazard ratio; CI, confidence intervals; ODA, objective data assessment; SGA, subjective global assessment; AGR, albumin to globulin ratio.
